# Immunocompromised Patients with Protracted COVID-19: a Review of “Long Persisters”

**DOI:** 10.1007/s40472-022-00385-y

**Published:** 2022-11-12

**Authors:** Veronica Dioverti, Sonsoles Salto-Alejandre, Ghady Haidar

**Affiliations:** 1 Division of Infectious Diseases, Johns Hopkins University, 600 N Wolfe St, Baltimore, MD 21287 USA; 2 Clinical Unit of Infectious Diseases and Microbiology, Institute of Biomedicine of Seville (IBiS)/Virgen del Rocío University Hospital/University of Seville/CSIC, Avda. Manuel Siurot s/n 41013, Seville, Spain; 3 Division of Infectious Diseases, University of Pittsburgh and UPMC, 3601 Fifth Ave, Falk Medical Building, Suite 5B, Pittsburgh, PA 15213 USA

**Keywords:** COVID-19, SARS-CoV2, Prolonged shedding, Prolonged COVID-19, Immunocompromised

## Abstract

**Purpose of Review:**

Certain immunocompromised individuals are at risk for protracted COVID-19, in which SARS-CoV-2 leads to a chronic viral infection. However, the pathogenesis, diagnosis, and management of this phenomenon remain ill-defined.

**Recent Findings:**

Herein, we review key aspects of protracted SARS-CoV-2 infection in immunocompromised individuals, or the so-called long persisters, and describe the clinical presentation, risk factors, diagnosis, and treatment modalities of this condition, as well as intra-host viral evolution. Based on the available data, we also propose a framework of criteria with which to approach this syndrome.

**Summary:**

Protracted COVID-19 is an uncharacterized syndrome affecting patients with B-cell depletion; our proposed diagnostic approach and definitions will inform much needed future research.

## Introduction: Defining the Syndrome and Its Risk Factors

Despite major advances in our understanding, treatment, and prevention of coronavirus disease 2019 (COVID-19), there continues to be a lack of consensus surrounding how to define and approach immunocompromised individuals with persistent and protracted severe acute respiratory syndrome coronavirus 2 (SARS‑CoV‑2) infection, also known as “long persisters.” This phenomenon should be distinguished from “long COVID-19” or a “post-COVID-19 condition,” which is defined by the World Health Organization as a syndrome which occurs in individuals (regardless of underlying host status) who contracted COVID-19 within the prior 3 months and have at least 2 months of ongoing symptoms (such as fatigue, dyspnea, and cognitive dysfunction), with no alternative diagnosis [1]. Since the literature describing long persisters consists almost entirely of case reports and case series, little can be concluded about the risk factors, natural history, and optimal treatment of these patients. Nonetheless, several case studies have shown compelling evidence of persistent infection, by demonstrating either successful isolation of infectious SARS-CoV-2 weeks to months after the initial infection [2, 3, 4•, 5–20], and/or intra-host evolution of virus with clonal relatedness to the initial infecting strain (thus ruling out superinfection) [2, 3, 4•, 5–10, 12–20, 21•, 22–32], often with evidence of absent humoral immunity despite protracted illness. Symptoms of these patients have varied from recurrent severe pneumonia [4•, 6, 8, 11, 24] to asymptomatic courses [2, 15, 23, 25]. To avoid confusion with long COVID-19 syndrome, we will refer to this condition as “protracted COVID-19/SARS-CoV-2 infection.”

The underlying host factors that predispose to protracted SARS-CoV-2 infection remain to be defined. The literature consists of a predominance of individuals with B-cell depletion from a range of conditions, such as hematological malignancies, Chimeric Antigen Receptor (CAR)-modified T-cell therapy, hematopoietic cell transplantation (HCT), or anti-CD20 monoclonal antibodies to treat rheumatologic and other conditions [2, 3, 4•, 5–11, 13, 14, 17, 18, 20, 21•, 22, 24–26, 28–30, 32–34]. A few reported cases provide compelling evidence of prolonged infection in solid organ transplant (SOT), AIDS, and/or other conditions [7, 9, 10, 12, 15, 16, 23, 27, 31]. However, these reports are limited by publication bias, with clinicians opting to only publish “interesting” and “noteworthy” cases. Thus, although it is biologically plausible that B-cell depletion may be the major risk factor for protracted SARS-CoV-2 infection as neutralizing antibodies are required for control of SARS-CoV-2 [35], any individual with severe immunodeficiency and COVID-19 should be considered at risk for protracted infection until larger studies are carried to better define this syndrome. In addition, a precise definition of severe immunodeficiency is difficult, as the net state of immunosuppression can only be approximated. Nonetheless, in order to help standardize the approach to defining protracted SARS-CoV-2 infection in an immunocompromised individual, and to differentiate the approach to these patients from that of patients with long COVID-19, we propose the preliminary framework outlined in Table 1. This framework relies on virologic criteria (i.e., the requirement of persistently positive SARS-CoV-2 PCR testing beyond 21 days), clinical and radiographic criteria, and host criteria. We underscore however that these criteria are based on case reports and case series and are expected to change over time as new evidence emerges and as our understanding of the biology of SARS-CoV-2 infection in immunocompromised individuals evolves.

**Table 1:** Proposed diagnostic criteria for protracted COVID-19 in immunocompromised hosts (long persisters)

Criteria	
Virologic	Persistently positive SARS-CoV-2 PCR ≥ 21 days
Clinical	Persistent/relapsing symptoms (fever, dyspnea, hypoxemia) after extensive negative infectious work up
Imaging	Persistent/relapsing changes on chest-X ray or CT scan after extensive negative infectious work up
Host	Underlying immunocompromise: HCT, CAR T-cell recipient CLL, DLBCL, other lymphoma, or B-cell malignancy SOT Anti-CD19/20 therapy or other B/T cell targeted therapies Primary and acquired immunodeficiencies

*HCT*, hematopoietic cell transplant; *CAR-T-cell*, chimeric antigen receptor-modified T-cell; *CLL*, chronic lymphocytic leukemia; *DLBCL*, diffuse large B-cell lymphoma (particularly if receiving B-cell depleting therapies); *SOT*, solid organ transplant

## Treatment Options

### Direct Acting Antivirals

Several antivirals have been approved for treatment of COVID-19. Remdesivir (RDV), an inhibitor of the viral RNA-dependent RNA polymerase, was approved for treatment of hospitalized patients >12 years of age in October 2020. In the ACTT-1 trial, hospitalized patients were randomized to 5 days of intravenous RDV or placebo [36]. There was a significant reduction in time to clinical recovery (11 days vs 15 days for placebo) and a non-significant trend toward decreased mortality (8% for RDV and 11.6% for placebo [36]). Diaz et al subsequently demonstrated a mortality benefit [37]. Duration of treatment with RDV varies from 3 to 10 days, showing benefits even with shorter 3-day courses provided it is initiated early during infection [38, 39].

Most data regarding the use of RDV for treatment of protracted COVID-19 come from case series or reports [22, 40, 41•, 42]. RDV monotherapy has been rarely described and is reported to be ineffective [4•]. Thus, RDV is often used in combination with passive humoral therapy. In some cases, RDV resulted in partial improvement and was then followed by convalescent plasma (CCP) infusion [43]. Brown et al. described 31 immunocompromised individuals with a median duration of symptoms of 62 days; all patients were considered “B-cell depleted” due to either a primary immunodeficiency leading to hypogammaglobulinemia or treatment with an anti-CD20 targeted drug. In total, 65% of patients (20/31) cleared the infection. Over half of the patients received a combination of RDV and an antibody-based therapy (monoclonal antibody [mAb] or CCP), whereas 7 patients received RDV monotherapy. None of the patients included received antibody-based monotherapy. The use of combination therapy was associated with increased odds of clearing the infection compared to RDV monotherapy (odds ratio of 23.1 (95% CI = 1.3–424.9 [*p*=0.035]) [41•]. Interestingly, the authors reported high rates of viremia in the subgroup of patients in whom it was measured (7/12 patients, 58.3%). Emergence of resistance has also been reported with RDV [44].

Molnupiravir and the combination of nirmatrelvir with ritonavir were granted emergency use authorization (EUA) by the US Food and Drug Administration (FDA) in December of 2021; neither of these agents has been used in immunocompromised individuals with protracted COVID-19.

### Passive Humoral Therapy

#### Convalescent Plasma

Literature regarding the benefits of CCP in the treatment of COVID-19 has been controversial, and data on immunocompromised individuals is scarce. A subgroup analysis of 126 immunodeficient patients from the REMAP-CAP Investigators showed a possible benefit [45]. Some investigators have used serial doses of CCP for treatment of protracted COVID-19 in patients with absent humoral immunity. Hueso el al. reported on 17 patients with B-cell depletion and/or hypogammaglobulinemia with protracted symptoms (median of 56 days) that responded to two transfusions of CCP [46•]. The vast majority experienced improvement in symptoms; moreover, SARS-CoV-2 RNAemia decreased within 7 to 14 days in all 9 patients in whom it was measured [46•].

Lang-Meli et al. evaluated 16 patients with primary immunodeficiencies treated with CCP between 8 and 132 days after onset of symptoms; about half of the patients received RDV. Treatment resulted in an increase in the cycle threshold (Ct) for SARS-CoV-2 PCR on nasopharyngeal (NP) swabs as well as symptomatic improvement in all patients [47]. The results of full-length SARS-CoV-2 genome sequencing were available in only 3 patients; emergence of a new viral subpopulation was noted in one patient who received a second course of CCP which ultimately resulted in viral clearance. As described in the “Direct Acting Antivirals” section, Brown et al suggested that combination of RDV and passive humoral therapy is superior to RDV alone for clearing protracted infections [41•]; however, Hueso et al. used CCP alone with success [46•]. Smaller case series and case reports have shown clinical improvement and/or viral clearance [48–53], while others have failed to show effectiveness [2, 6].

#### Monoclonal Antibodies

Another approach for the management of suspected protracted SARS-CoV-2 infection is the use of monoclonal antibodies (mAb). In some cases, treatment courses of RDV and/or CP failed to clear the virus, resulting in the use of different mAb such as sotrovimab [54]and casirivimab/imdevimab [26, 55, 56]. There have been reports of bamlanivimab use, but rapid development of resistance did not allow further use as monotherapy [25, 28, 33, 57].

Unfortunately, the use of mAb in the USA is limited by the criteria defined in the FDA’s EUA document, which has generally restricted their use for treating COVID-19 only among non-hospitalized individuals who do not require supplemental oxygen support, and who are within 7–10 days of their diagnosis (FDA fact sheet). Thus, clinicians in the USA are unable to deviate from these criteria, unless the manufacturer of the drug offers a compassionate use program, where the drug can be used outside the EUA on a case-by-case basis. Additionally, the emergence of new variants has resulted in resistance to all prior mAb, such that only two are in current use, one for treatment within 7 days of symptom onset (bebtelovimab) and another one for pre-exposure prophylaxis (tixagevimab-cilgavimab).

### Other Combination Therapy

Combination antiviral therapy may have theoretical advantages. Indeed, using drugs that bind to multiple viral targets may aid in viral clearance, similarly to HIV and HCV. In vitro studies have shown increased antiviral activity when combining nirmatrelvir/ritonavir with remdesivir and molnupiravir as well as with molnupiravir-based regimens [58, 59]; however, none of these regimens has been tested in humans. These combinations may be particularly helpful in individuals with protracted COVID-19, as they may prevent emergence of resistance and/or new variants. Intravenous immunoglobulin (IVIg) replacement has been occasionally used in combination with RDV and/or CCP [8, 60, 61]

### SARS-CoV2-Specific T-Cells

Although neutralizing antibodies are required for control of SARS-CoV-2 infection [35], preliminary data suggest that T-cells may be able to protect against mAb-resistant SARS-CoV-2 variants, even in the absence of a neutralizing antibody response [62]. Additionally, Spike proteins from novel variants may not escape T-cell-mediated immunity elicited by the wild-type S protein [62]. Thus, there is interest in utilizing banked or “off-the-shelf” SARS-CoV-2 viral-specific T-cells (VSTs) for the treatment of COVID-19 in immunocompromised individuals. There is indeed precedent supporting this approach with other intractable viral infections, such as Epstein-Barr virus (EBV), cytomegalovirus (CMV), and adenovirus in HCT recipients [63], CMV infection in SOT recipients [64], and even progressive multifocal leukoencephalopathy in a small case series of individuals with varied causes of immunodeficiency [65]. The results of these and other studies, while promising, are also mixed, with some patients responding to VSTs and others exhibiting no response, and with multiple lingering unknowns such as optimal dosing and frequency of administration, the durability of VSTs in an immunodeficient host (particularly those receiving iatrogenic immunosuppression which is expected to dampen the activity of VSTs), and the underlying biology of treatment successes versus failures.

In the only published case report of SARS-CoV-2 VSTs to date, investigators described a heart transplant recipient with refractory COVID-19 pneumonia, whose condition progressed despite remdesivir, corticosteroids, and tocilizumab [66]. The patient subsequently received three doses of an off-the-shelf partially HLA matched SARS-CoV-2 VST (2 × 10^7^ cells per dose, approximately 14 ± 4 days apart), which resulted in eradication of the virus and clinical remission. A recent presentation at the American Transplant Congress meeting suggested a potential role of SARS-CoV-2 VSTs in six patients with hematologic malignancies or SOT, though the results were mixed [67].

Although there is much to be learned about the efficacy of SARS-CoV-2 VSTs, including issues related to cost, manufacturing, access, HLA-matching, and patient candidacy, administration of VSTs does appear to be safe, with newer products demonstrating either no safety concerns or only minimal grade 1 graft-versus-host disease in HCT [68]. Since many VST products appear to be long-lasting, with some VSTs being detectable for as long as 9 years after infusion [68], whether SARS-CoV-2 VSTs can be used for primary prophylaxis of COVID-19 should be evaluated in clinical trials. Indeed, because immune escape of SARS-CoV-2 from T-cells has not been a major phenomenon [69] (in contrast immune escape of novel variants from mAb), the use of VSTs for COVID-19 prophylaxis may circumvent the issue of emergence of SARS-CoV-2 variants with reduced susceptibility to other prophylactic interventions such as monoclonal antibodies [70]. However, whether prophylactic VSTs will persist in the absence of ongoing viral stimulation is unknown.

### Other

A single case report exists of therapeutic vaccination with the BNT162b2 mRNA vaccine in a patient with Wiskott-Aldrich syndrome. The authors demonstrated enhanced cellular responses and seroconversion after vaccination, with viral clearance 72 days after the first vaccine dose [71]. A new COVID-19 peptide vaccine that induces T-cell immunity is being evaluated in patients with B cell or antibody deficiency; an open-label phase I trial showed potent T-cell responses and a favorable safety profile in healthy adults [72]. However, peptide-based COVID-19 vaccines have not been used in protracted infections.

Interferon gamma has been proposed as therapy to stimulate cellular immunity against viral infections. Van Laarhoven et al. used this approach in 5 patients with COVID-19; most had previously received RDV, CP, or IVIg [73]. Four patients cleared SARS-CoV-2, and one patient passed away after a prolonged hospital stay after. Another case was successfully treated with interferon beta-1b in combination with RDV and IVIg [74].

## Role of Cycle Threshold Values and Viral Loads to Guide Management

### Cycle Threshold

Understanding the level of infectivity in patients with prolonged SARS-CoV-2 nucleic acid test positivity using a quantitative test result is crucial, to guide both therapy and public health policy, as immunocompromised individuals may remain test-positive and infectious for weeks and thus contribute to ongoing community spread of the virus [75]. The SARS-CoV-2 reverse transcriptase quantitative PCR (RT-qPCR) test provides a quantitative value, the cycle threshold (Ct) value, which inversely correlates with viral loads [76]. Determining a correlation between Ct values and replication-competent virus may help distinguish between infectious virus and non-viable virus which is no longer infectious. Although many reports have demonstrated a correlation between decreases in Ct values with recurrence of symptoms and culture positivity in immunocompromised individuals [2, 3, 4•, 11], the use of Ct values in the clinical setting is not currently endorsed by the Infectious Diseases Society of America (IDSA) or the Association of Molecular Pathology due to lack of standardization and discrepant results [77]. A standardized quantitative test is urgently needed to help differentiate infectious versus non-viable virus, and perhaps to guide treatment.

### Viral Load

It is now becoming increasingly understood that plasma SARS-CoV-2 viremia correlates with the severity of pneumonia. In one study, SARS-CoV-2 viral RNA was detected in plasma of 100%, 52.6%, and 11.1% of individuals in the intensive care unit (ICU), regular floor, and outpatient setting, respectively [78], and plasma viral RNA levels were significantly higher in ICU patients than in non-ICU patients and correlated with higher disease severity scores. Plasma SARS-CoV-2 RNA also served as a biomarker for disease severity, with levels > 6000 copies/mL being strongly associated with mortality. Furthermore, it appears as though plasma RNA levels also correlate with lower respiratory tract SARS-CoV-2 RNA levels [79], suggesting that it may have a role as a surrogate for overall SARS-CoV-2 disease burden. In another study, plasma SARS-CoV-2 RNA levels at the time of presentation predicted both severe disease and 28-day mortality [80], with upregulation of prominent proteomic pathways such as those related to SARS-COV-2 entry, tissue damage, and coagulation pathways. High plasma SARS-CoV-2 RNA levels have not surprisingly also been reported in immunocompromised individuals with COVID-19 [4•, 6, 8, 28].

Although measurement of plasma viral loads is not clinically available, its use as a surrogate endpoint for COVID-19 outcomes in immunocompromised individuals should be evaluated in clinical trials. Given that we are now in the era of direct-acting antivirals that target SARS-CoV-2, it is imperative to determine whether treating immunocompromised individuals with antivirals (such as RDV, nirmatrelvir/ritonavir, and molnupiravir) until suppression of their plasma SARS-CoV-2 viremia will improve clinical outcomes and prevent protracted infection compared to standard practice whereby all individuals receive a fixed duration of these drugs. A precedent for such an approach already exists with other opportunistic viruses such as cytomegalovirus [81], in which durations of therapy vary based on the viral response to antivirals.

## Emergence of Variants

SARS-CoV-2 evolution may also develop in an immunocompromised individuals, resulting in emergence of mutations within the Spike gene and other areas of the genome [2, 3, 4•, 5–10, 12, 15–17, 19, 20, 21•, 22, 25–32, 82]. Indeed, it is speculated that many of the circulating variants of concern may have emerged as a result of intra-host evolution in immunocompromised individuals with protracted infection, followed by spread to the general population [6, 83]. In one case report, a CAR-T-cell recipient with over 70 days of active SARS-CoV-2 infection exhibited the evolution of mutations in the Spike gene (namely a Y144 deletion and a D215G substitution) that would eventually be identified months later in what were then referred to as the UK (alpha) and South African (beta) variants, long after the patient’s death [6]. Other case reports and case series have also demonstrated the simultaneous existence of multiple mixed populations of SARS-CoV-2 variants that emerged in the setting of severe immunosuppression [24, 27], or of mutations in the Spike gene (such as E484Q) resulting in reduced susceptibility to monoclonal antibodies such as bamlanivimab [28, 33].

Immune escape may also develop during the course of natural infection in the certain hosts. For example, in a patient with advanced HIV infection (CD4 count = 6 cells/uL) and over 190 days of COVID-19 [12], the patient’s original infecting SARS-CoV-2 strain developed mutations found in Omicron and other variants and showed evidence of immune escape against self-plasma, Pfizer BNT162b2, and antibodies elicited by Delta. Of additional concern is a recent report demonstrating treatment-emergent mutations in the Spike gene conferring resistance to sotrovimab that developed within 2 weeks of therapy in 4 immunocompromised individuals (lung transplant, common variable immunodeficiency, kidney transplant, and myelodysplasia) [16].

## Hospital Precautions and Self-quarantine: When Is It Safe to Discontinue?

It is well-established that healthy people generally do not harbor infectious virus beyond 10 days of initial infection [84]. Additionally, although SARS-CoV-2 RNA from healthy individuals may be detected for up to 28 days or longer [85], this phenomenon represents the identification of inert, non-infectious, non-replicating RNA fragments [84]. Thus, the CDC recommends quarantine for at least 5 days for otherwise healthy individuals, while wearing a mask in public for a total of 10 days [86].

However, hospital systems continue to struggle with the optimal approach for infection prevention practices among immunocompromised individuals with COVID-19 due to the lack of readily available or accurate diagnostic modalities that can distinguish between infectious virus and non-viable RNA with a high degree of certainty. Practice guidance from the CDC regarding this issue has evolved over time, with the most recent recommendation suggesting a composite approach utilizing a PCR test-based strategy, beginning at 20 days after the initial positive SARS-CoV-2 test, with consultation with an infectious disease specialist [87]. This contrasts with transmission-based recommendations for other hosts, in whom a time-based strategy (generally up to 10 days since symptom onset) is employed. Although this approach is easily implemented, it may result in some immunocompromised individuals being isolated for longer than is necessary, given the difficulty in determining whether a positive PCR test represents infectious virus or not. Further complicating this matter is the phenomenon of intermittent SARS-CoV-2 PCR positivity, which has been demonstrated in immunocompromised individuals with protracted courses [3, 4•].

Although it appears that low Ct values (generally < 25) may correlate with infectivity [88], these values are not recommended for clinical use in the USA, and isolating SARS-CoV-2 by culture is cumbersome, expensive, and not readily available. Recently, it has been suggested that SARS-CoV-2 antigen positivity correlates better with infectious virus than does PCR positivity [88]. Whether the use of SARS-CoV-2 antigen testing in immunocompromised individuals can streamline infection prevention practices such that those with negative antigen results are designated “non-infectious” remains to be determined. Until more data are available, clinical judgement and an understanding of the behavior of SARS-CoV-2 in immunocompromised individuals are recommended when devising local infection prevention policies. An example of what may be considered is outlined in Table 2, although it is understood that these suggestions should not be taken as formal recommendations but rather as a framework with which institutions struggling to cope with infection prevention policies may approach this problem. It is also expected that these policies may shift depending on the stage of the pandemic and bed capacity, and that they will need to be reappraised as new data emerge.

**Table 2:** Proposed criteria to be considered for stratifying risk of infectivity of symptomatic immunocompromised patients with COVID-19. These criteria should not be considered guidelines and should be subject to modification with the emergence of new data and based on hospital bed capacity

Degree of immunocompromise	Infection prevention approach
Moderate^a^	Discontinue precautions 24 h after resolution of fever (without antipyretics), improvement of symptoms, and at least 20 days since first positive test
Severe^b^	Consider test-based strategy using PCRs under infectious disease specialist guidance Discontinue precautions with upon negative PCR result^c^

^a^Immunocompromised, moderate: immunocompromising conditions not classified as severe, including (but not limited to) chemotherapy for cancer, untreated HIV infection with CD4 T lymphocyte count < 200, combined primary immunodeficiency disorder, and receipt of prednisone > 20 mg/day for more than 14 days

^b^Immunocompromised, severe: hematologic malignancy on treatment; hematopoietic cell transplant or CAR-T-cell therapy within the preceding 6 months, or with systemic therapy for acute GVHD in the past 6 months; solid organ transplant (SOT) within the preceding 6 months; SOT or heme malignancy receiving treatment with specific immunocompromising therapies (thymoglobulin, alemtuzumab, fludarabine, cladribine, or anti-CD20 monoclonal antibodies) in the preceding 6 months; other serious T- and/or B-cell deficiencies determined on a case-by-case basis that may include untreated HIV with CD4 T lymphocyte count < 100, rituximab therapy, and select primary immunodeficiency disorders

^c^Some centers may favor 2 negative PCR results given reports of intermittent shedding of infectious virus

## When to Proceed with Immune/Chemotherapy and Transplantation

Decisions around when to proceed with further immunotherapy, chemotherapy, or transplantation should consider the risks of progression of the underlying condition while avoiding additional immunosuppression during active COVID-19, versus further decreasing immune responses that are crucial to containing/eradicating viral infections. Urgent transplantation can be a life-saving procedure, and timing of organ donation is difficult to predict. Similarly, prolonged delays in CAR-T-cell therapy or HCT may not be feasible in certain hematologic malignancy patients, who may die as a result of their underlying malignancy. A thoughtful and thorough discussion among health care personnel and the patient (and/or patient’s proxy) should be held prior to any procedure that further weakens immune responses, as it could lead to worsening SARS-CoV-2 infection with devastating consequences.

Emerging data suggests that it might be feasible to proceed with immune/chemotherapy in some circumstances where a multidisciplinary team with expertise in these highly vulnerable patients exist, as well as non-lung solid organ transplant [89, 90]. However, all these experiences and recommendations are based on acute COVID-19 in otherwise healthy donors or in recovered candidates, and the optimal timing to proceed with immunosuppressive treatments for patients with protracted COVID-19 is unknown. We refer the reader to professional societies recommendations, which are frequently updated (AST COVID-19 resources, OPTN network, ASTCT COVID-19 resource community, ASH COVID-19 resources).

## Future Directions

The morbidity and mortality associated with COVID-19 have decreased since the onset of the pandemic, largely due to widespread vaccination and safe and effective treatments, as well as the progress that has been achieved in our general understanding of the biological aspects of SARS-CoV-2 infection. However, several key questions remain about immunocompromised individuals with protracted COVID-19. It is imperative for the scientific community to define the prevalence of protracted infection and the risks factors that predispose to this condition. It is also critical to improve our understanding of intra-host viral evolution and the emergence of novel variants, as this may have a tremendous impact on public health. Finally, it is of paramount importance to optimize the treatment of these patients, in whom the virus behaves not as an acute but rather as a chronic viral infection. To accomplish this, the scientific community must conduct trials that both enhance our knowledge and either lead to newer therapies or to the use of combinations of existing drugs with different mechanisms of action that are given until remission of infection. It is hoped that these approaches will reduce COVID-19-related morbidity and mortality in these patients and allow them to safely proceed with life-saving immunosuppressive treatments that they ultimately need.
